# Metabolic reprogramming and macrophage expansion define ACPA-negative rheumatoid arthritis: insights from single-cell RNA sequencing

**DOI:** 10.3389/fimmu.2024.1512483

**Published:** 2025-01-03

**Authors:** Yafeng Jiang, Zhaolan Hu, Roujie Huang, Kaying Ho, Pengfei Wang, Jin Kang

**Affiliations:** ^1^ Department of Hematology, the Second Xiangya Hospital of Central South University, Changsha, China; ^2^ Department of Anesthesiology, The Second Xiangya Hospital, Central South University, Changsha, China; ^3^ Department of Obstetrics and Gynecology, National Clinical Research Center for Obstetric & Gynecologic Diseases, Peking Union Medical College Hospital, Chinese Academy of Medical Sciences and Peking Union Medical College, Beijing, China; ^4^ School of Nursing, The Hong Kong Polytechnic University, Hong Kong, Hong Kong SAR, China; ^5^ Department of Rheumatology and Immunology, the Second Xiangya Hospital of Central South University, Changsha, China; ^6^ Department of Rheumatology and Immunology, Clinical Medical Research Center for Systemic Autoimmune Diseases in Hunan Province, Changsha, China

**Keywords:** rheumatoid arthritis, single-cell RNA sequencing, ACPA, synovial macrophage, beta-alanine and glutathione metabolism

## Abstract

**Background:**

Anti-citrullinated peptide antibodies (ACPA)-negative (ACPA−) rheumatoid arthritis (RA) presents significant diagnostic and therapeutic challenges due to the absence of specific biomarkers, underscoring the need to elucidate its distinctive cellular and metabolic profiles for more targeted interventions.

**Methods:**

Single-cell RNA sequencing data from peripheral blood mononuclear cells (PBMCs) and synovial tissues of patients with ACPA− and ACPA+ RA, as well as healthy controls, were analyzed. Immune cell populations were classified based on clustering and marker gene expression, with pseudotime trajectory analysis, weighted gene co-expression network analysis (WGCNA), and transcription factor network inference providing further insights. Cell-cell communication was explored using CellChat and MEBOCOST, while scFEA enabled metabolic flux estimation. A neural network model incorporating key genes was constructed to differentiate patients with ACPA− RA from healthy controls.

**Results:**

Patients with ACPA− RA demonstrated a pronounced increase in classical monocytes in PBMCs and C1QChigh macrophages (p < 0.001 and p < 0.05). Synovial macrophages exhibited increased heterogeneity and were enriched in distinct metabolic pathways, including complement cascades and glutathione metabolism. The neural network model achieved reliable differentiation between patients with ACPA− RA and healthy controls (AUC = 0.81). CellChat analysis identified CD45 and CCL5 as key pathways facilitating macrophage-monocyte interactions in ACPA− RA, prominently involving iron-mediated metabolite communication. Metabolic flux analysis indicated elevated beta-alanine and glutathione metabolism in ACPA− RA macrophages.

**Conclusion:**

These findings underscore that ACPA-negative rheumatoid arthritis is marked by elevated classical monocytes in circulation and metabolic reprogramming of synovial macrophages, particularly in complement cascade and glutathione metabolism pathways. By integrating single-cell RNA sequencing with machine learning, this study established a neural network model that robustly differentiates patients with ACPA− RA from healthy controls, highlighting promising diagnostic biomarkers and therapeutic targets centered on immune cell metabolism.

## Introduction

Rheumatoid arthritis (RA) is a chronic autoimmune disorder marked by persistent synovial inflammation, leading to joint destruction and impaired functionality. Its pathogenesis is driven by a multifaceted interaction of genetic, environmental, and immunological factors that promote immune dysregulation and chronic synovial inflammation (1, 2). A central feature of RA is the presence of autoantibodies, notably anti-citrullinated peptide antibodies (ACPA), which exhibit high specificity for the disease and serve as important diagnostic and prognostic markers (3, 4). Patients with ACPA-positive (ACPA+) RA typically experience a more aggressive disease course, characterized by accelerated joint damage and systemic involvement (5).

Nevertheless, approximately 20–30% of patients with RA are ACPA-negative (ACPA−), lacking these specific autoantibodies (6). ACPA− RA presents distinct clinical challenges, as it may follow unique disease trajectories and exhibit variable therapeutic responses compared to ACPA+ RA (7). The absence of ACPA complicates early diagnosis, potentially delaying treatment onset and impacting long-term patient outcomes (8). Furthermore, the immunopathological mechanisms underlying ACPA− RA remain incompletely characterized, posing a barrier to the development of targeted treatments for this subgroup (9).

Recent findings suggest that ACPA− RA represents a distinct clinical entity with unique immunological characteristics (10). Variations in genetic predisposition, cytokine profiles, and immune cell composition differentiate ACPA− RA from its ACPA+ counterpart (11, 12). Notably, alterations in monocyte and macrophage populations have been implicated in RA pathogenesis (13). Monocytes and macrophages are pivotal in inflammation and immune modulation, driving synovial hyperplasia and joint destruction through the release of pro-inflammatory cytokines and matrix-degrading enzymes (14). However, the precise roles of these immune cells in ACPA− RA remain inadequately elucidated.

Metabolic reprogramming in immune cells is increasingly recognized as a pivotal factor in autoimmune diseases, including RA (15). During immune activation, differentiation, and effector functions, immune cells reconfigure their metabolic pathways to meet heightened energetic and biosynthetic demands (16). Dysregulated metabolic processes can profoundly impact immune cell function, fostering chronic inflammation (17). In RA, research has demonstrated that altered glucose and lipid metabolism in both synovial fibroblasts and immune cells accelerates disease progression (15, 18). However, the metabolic characteristics of immune cells in ACPA− RA remain largely unexamined.

Advancements in single-cell RNA sequencing (scRNA-seq) now enable precise profiling of cellular heterogeneity, facilitating the identification of novel cell subtypes and disease-associated pathways (19). Utilizing scRNA-seq on peripheral blood mononuclear cells (PBMCs) and synovial tissue mononuclear cells (STMCs) from patients with RA allows researchers to delineate the complex cellular interactions and metabolic pathways underlying inflammation (20). Coupling scRNA-seq data with computational models further supports the estimation of metabolic fluxes and the construction of cell-cell communication networks (21).

This study investigates the cellular composition, metabolic reprogramming, and intercellular communication specific to ACPA− RA. scRNA-seq analysis was performed on PBMCs and STMCs from both patients with ACPA− RA and those with ACPA+ RA, with a focus on monocyte and macrophage subsets. Our hypothesis posits that patients with ACPA− RA exhibit distinctive immune cell profiles and metabolic pathways that underlie their unique clinical features. By identifying differentially expressed genes, metabolic modules, and signaling pathways, this research aims to pinpoint potential biomarkers and therapeutic targets for ACPA− RA. Our findings offer new insights into ACPA− RA pathogenesis and underscore the critical role of metabolism in modulating immune responses within this patient subgroup.

## Methods

### Data acquisition

The sequence data used for this study have been deposited in the Genome Sequence Archive at the BIG Data Center, Beijing Institute of Genomics (BIG), Chinese Academy of Sciences, under accession code HRA000155 (22). Researchers seeking access must submit an application for approval to utilize this dataset for further analysis.

### Single-cell RNA sequencing alignment and quality control

Raw 10x Genomics sequencing data were processed with CellRanger v2.2.0 using the human transcriptome GRCh38-1.2.0 as a reference (23). Additional quality control measures were applied to remove low-quality cells, specifically excluding cells with mitochondrial gene expression exceeding 5%. Single-cell read counts from all samples were analyzed with the Seurat package (v5.0.1) in R (v4.3.1), where data were transformed into Seurat objects (24). Filtering criteria included retaining cells with unique molecular identifier (UMI) counts between 1000 and 25000 and genes detected in at least five cells while restricting cells to those expressing between 500 and 3500 genes. Post-filtering, data normalization was executed with Seurat’s NormalizeData function, followed by the identification of highly variable genes using FindVariableFeatures.

### Integration of scRNA-seq data from the same tissue

For tissue-specific scRNA-seq data integration (PBMC or synovial tissue), the Harmony package was employed. Downstream analyses, including dimensionality reduction and clustering, leveraged highly variable gene correlations.

### Dimensionality reduction and major cell type annotation

Separate analyses were conducted for PBMC and synovial tissue datasets, with adjustments for confounders such as UMI counts, mitochondrial gene percentage, and cell cycle genes. Gene expression was scaled to unit variance, and dimensionality was reduced using principal component analysis (PCA), selecting the top 20 principal components (PCs) based on the elbow plot and variance explained. Cell clusters were visualized in two-dimensional space *via* Uniform Manifold Approximation and Projection (UMAP), and unsupervised clustering was executed with Seurat’s FindClusters function, applying the Louvain algorithm for community detection. Resolution parameters were set to 0.5 for PBMC and 0.8 for synovial tissue.

**Figure 1:**
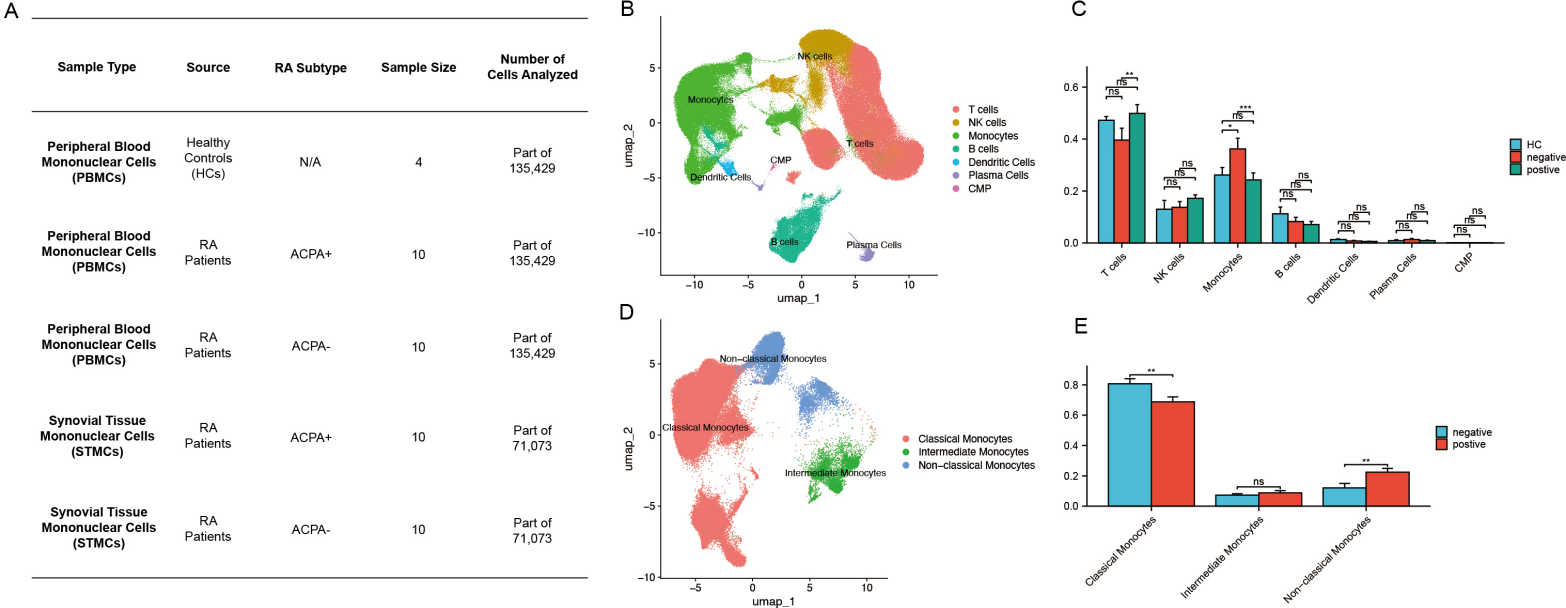
**(A)** Sample size distribution of scRNA-seq data from HC, ACPA+ RA, and ACPA− RA individuals. **(B)** UMAP clustering of immune cells from PBMCs. **(C)** Wilcoxon test comparing immune cell proportions between ACPA+ and ACPA− groups. **(D)** UMAP clustering of monocyte subpopulations. **(E)** Wilcoxon test comparing monocyte subpopulations between ACPA+ and ACPA− groups. (P-values are expressed as follows: * p ≤ 0.05, ** p ≤ 0.01, and *** p ≤ 0.001.).

Resolution settings were determined through an iterative approach, evaluating cluster stability and biological significance by varying resolution from 0.2 to 1.5 in 0.2 increments. Silhouette scores and modularity metrics were utilized to assess cluster cohesion and separation. The final resolutions provided an optimal balance, capturing distinct subpopulations without excessive clustering of biologically similar cells. Cell identities were assigned based on known marker genes for each cell type, as illustrated in **Figure 1A** and **Supplementary Figure 1B**, with validation through cross-referencing published datasets and established cell type annotations. For ambiguous marker expression, differential expression analysis was applied to confirm cell identity.

### Differential expressed genes and pathway analysis

Differentially expressed gene (DEG) analysis was conducted using the FindMarkers function in Seurat with the Wilcoxon test. Bonferroni correction was applied to adjust p-values, and DEGs were filtered at a significance threshold of p < 0.05. For this study, the mini pct was set to 0.1, meaning at least 10% of cells in either group must express the gene for it to be included in the analysis. Enrichment analysis of DEGs was carried out using the clusterProfiler package (v3.12.0), examining Gene Ontology (GO) terms and Kyoto Encyclopedia of Genes and Genomes (KEGG) pathways (25). Specific parameters used in the analysis include a p-value cutoff of 0.05, a q-value cutoff of 0.2, and a gene set size range of 10 to 500. Multiple testing correction was performed using the Benjamini-Hochberg method. These parameter choices were guided by established practices to ensure biologically meaningful and statistically reliable results. To elucidate the functional roles of each macrophage subset, gene set variation analysis (GSVA) was performed with standard settings in the GSVA R package (v1.32.0).For this single-cell analysis, log-normalized expression data from Seurat were used as input. Pathways were selected from the MSigDB KEGG gene set collection, ensuring a comprehensive evaluation of biological processes. Specific parameters for the gsva() function included method = “gsva” (default kernel-based density estimation), mx.diff = TRUE (to calculate enrichment scores based on maximum difference between conditions), and a min.sz = 10 and max.sz = 500 to ensure only biologically relevant pathways were considered while accounting for sparsity in single-cell datasets. These parameter choices were optimized for single-cell data to maintain robustness and biological interpretability.

Additionally, AUCell analysis was performed to evaluate the activity of gene sets in individual cells, complementing GSVA results by providing cell-level resolution. This analysis used the AUCell package (v1.12.0) with the AUCell_buildRankings() function to rank genes based on expression levels across individual cells. The AUCell_calcAUC() function was then applied to calculate the Area Under the Curve (AUC) scores for predefined gene sets, with thresholds determined based on empirical distributions. Parameters included a ranking threshold of 5% and the use of log-normalized data to ensure compatibility with single-cell datasets. These details enhance the transparency and reproducibility of the methods used in this study.

### Trajectory inference

The Monocle2 algorithm was applied to explore differentiation trajectories within selected clusters (26). Cells of interest were subset using the Seurat subset function, and a CellDataSet object was generated with Monocle2’s newCellDataSet function, setting the lowerDetectionLimit parameter to 0.5. Low-quality cells and genes were removed with min_expr = 0.1, and dimensionality reduction was conducted using the DDRTree method. Visualization of trajectories was achieved through plot_cell_trajectory and plot_genes_in_pseudotime functions.

### SCENIC analysis

To identify regulons (transcription factors [TFs], their modules, and potential targets) and assess their activity, this study employed the single-cell regulatory network inference and clustering (SCENIC) approach (27). This workflow began with the inference of co-expression modules using GRNBoost2, followed by motif pruning with cisTarget. Regulon activity was quantified with AUCell scores, and TF activity was evaluated using the Python-based tool pySCENIC (28). Leveraging the cis-target and motif databases, all TFs with motifs were analyzed to identify cell-type-specific regulons with high regulon specificity scores (RSS) (29).

### HdWGCNA analysis

High-dimensional weighted gene co-expression network analysis (hdWGCNA) was employed to identify key macrophage-related genes (30). Monocyte and macrophage populations were extracted from scRNA-seq data, gene expression correlation matrices were computed, and gene co-expression modules were identified. Critical parameters were carefully optimized during the hdWGCNA process to ensure robust network construction and module detection. In the initial step, gene expression correlation matrices were calculated, and the soft-thresholding power was determined to optimize scale-free network topology. A soft-thresholding power of 7 was selected to ensure that the network exhibited scale-free properties, a hallmark of biological networks. This selection was guided by plotting the scale-free topology model fit against various power values and choosing the point where the network’s R-squared value reached a plateau. Following network construction, co-expression modules—clusters of genes with similar expression patterns across the macrophage population—were identified. The relevance of these modules was assessed *via* module-trait relationship analysis, correlating each module with specific traits related to macrophage activation and inflammation. For each trait-related module, hub genes—genes with high intramodular connectivity central to the network structure—were identified. Hub genes were defined based on their connectivity scores (kME values) within their respective modules, following the approach outlined in previous studies.

### Deep neural network construction

A deep neural network (DNN) was constructed using PyTorch to define and optimize the network architecture. Based on PBMC expression data from 20 DEGs and corresponding cell-type data, the DNN was developed to distinguish patients with ACPA− RA from healthy controls (HC). Data were divided into a 70% training set and a 30% test set, with training performed over 1000 epochs using mini-batch gradient descent. The DNN architecture consisted of an input layer with 21 features, followed by four hidden layers containing 128, 64, 32, and 16 neurons, each employing Sigmoid activation functions, and concluded with a single Sigmoid neuron in the output layer for binary classification (ACPA-negative or healthy).In clinical settings, the characterization of macrophage populations plays a crucial role in diagnosing and understanding rheumatoid arthritis (RA) subtypes. If over 50% of a patient’s macrophages are found to be ACPA-negative, this could strongly suggest an ACPA-negative RA diagnosis. Otherwise, the patient is likely classified as healthy.

To prevent overfitting, early stopping was applied based on validation loss, and each hidden layer included a dropout rate of 0.2. Key model parameters, including learning rate, number of layers, and dropout rates, were optimized *via* grid search, exploring learning rates from 0.001 to 0.01. A learning rate of 0.005 was ultimately selected based on improved validation accuracy. Model performance was assessed through accuracy metrics and ROC curve analysis, with the ROC curve generated using Scikit-learn’s roc_curve function. Additionally, cross-validation was implemented to reinforce model robustness, averaging performance metrics across five folds to ensure generalizability.

### Cell communication and signaling pathways

Cell communication analysis was performed using the CellChat package in R with default parameters, focusing on PBMC monocyte and synovial macrophage subsets independently (31). The analysis utilized the human CellChatDB and enabled a comparative assessment of interactions between ACPA+ and ACPA− macrophage subpopulations and PBMC monocytes.

### MEBOCOST analysis

MEBOCOST, a Python-based tool, inferred metabolite-mediated cell communication from scRNA-seq data. This tool, which leverages a curated database of metabolite sensors and partners, identified sender and receiver cells based on metabolite outflow/inflow rates and enzyme/sensor expression levels. scRNA-seq expression data were first loaded into a Python pandas DataFrame, integrated with cell annotations, and then used to infer metabolic communications. Results were visualized to illustrate communication events, sender-to-receiver flows, and sensor expression levels.

### Construction of single-cell metabolic flux curves

Single-cell metabolic flux profiles were derived using the single-cell flux estimation analysis (scFEA) algorithm, a graph neural network-based approach (21). The algorithm utilized 168 metabolic modules, obtained from scFEA’s official GitHub repository (https://github.com/changwn/scFEA). KEGG enrichment analysis was conducted on input and output modules using MetaboAnalyst (https://www.metaboanalyst.ca/home.xhtml).

### Statistical analysis

All statistical analyses were performed using R software (v4.3.1), with visualizations generated in R Studio. Data were pre-processed to meet the assumptions for each statistical test, and appropriate transformations were applied when necessary. Statistical tests were selected based on data distribution and study design. For comparisons between two groups with normally distributed data and equal variances, Student’s t-test was used. The Wilcoxon Rank-Sum Test was applied for non-parametric data, providing a robust method for comparing medians between two independent groups, especially suitable for small sample sizes or skewed distributions. The Kruskal-Wallis Test was employed for comparisons across more than two independent groups with non-parametric data. To control the family-wise error rate, p-values were adjusted using the Holm-Bonferroni method. Statistical significance was set as follows: “ns” for p > 0.05, * for p ≤ 0.05, ** for p ≤ 0.01, *** for p ≤ 0.001, and **** for p ≤ 0.0001.

## Results

### Identification of distinct immune cell types in patients with RA

Single-cell sequencing data of immune cells from patients with ACPA− RA and patients with ACPA+ RA were obtained from the Genome Sequence Archive at the Big Data Center, Beijing Institute of Genomics, Chinese Academy of Sciences. The dataset comprised 44 samples, including CD45+ PBMCs isolated from HC (n = 4) and from ACPA+ (n = 10) and ACPA− (n = 10) RA individuals (**Figure 1A**). Additionally, synovial tissue mononuclear cells (STMCs) were obtained from ACPA+ (n = 10) and ACPA− (n = 10) RA individuals (**Figure 1A**). None of the patients were receiving disease-modifying antirheumatic drugs (DMARDs), corticosteroids, or targeted therapies at the time of sampling, though some opted for physical therapies, such as thermotherapy or acupuncture, to manage pain. A graph-based unsupervised clustering method was applied to identify cell types by examining typical marker genes. Cell populations identified included T cells, B cells, monocytes, dendritic cells, plasma cells, NK cells, and common myeloid progenitors (CMP). Each cell type was annotated according to well-characterized marker genes (**Figure 1B**, **Supplementary Figure 1A**). Specifically, T cells were defined by high expression of CD3D and CD3E, while NK cells were distinguished by NKG7 and GNLY. Monocytes were annotated by CD14 and FCGR3A, and dendritic cells by ITGAX and HLA-DQA1. B cells and plasma cells were characterized by distinct marker profiles, with B cells expressing CD19, MS4A1, and CD79A, and plasma cells marked by SDC1 and MZB1. CMPs were identified using CD34, KIT, and FLT3, established indicators of progenitor populations. Marker selection was based on specificity for each cell type, validated by previous research in the field. This rigorous marker selection and clustering approach enabled robust and precise classification of cell types within the dataset.

### Increased monocyte proportions in patients with ACPA− RA

The Wilcoxon test was applied to assess differences in immune cell type proportions across ACPA-positive, ACPA-negative, and HC groups. Results indicated a statistically significant increase in monocyte proportions within the ACPA-negative group compared to both ACPA-positive and HC groups (p < 0.001). Additionally, a significant difference was detected in T cell proportions between ACPA-negative and ACPA-positive groups (p < 0.01). No significant differences were observed for NK cells, B cells, dendritic cells, plasma cells, or CMPs across the groups (**Figure 1C**).

### Identification of monocyte subpopulations

Further dimensionality reduction and clustering analysis of monocytes identified three distinct subpopulations: classical, non-classical, and intermediate monocytes (**Figure 1D**). Classical monocytes were characterized by CD14 expression, non-classical monocytes by CD16, and intermediate monocytes by the co-expression of CD14 and CD16 (**Supplementary Figure 1B**).

### Patients with ACPA+ RA show increased classical monocytes and reduced non-classical monocytes

The Wilcoxon test was subsequently conducted to compare the proportions of monocyte subpopulations between ACPA-positive and ACPA-negative groups. This analysis revealed a statistically significant reduction in the proportion of non-classical monocytes in the ACPA-positive group relative to the ACPA-negative group (p < 0.01) (**Figure 1E**). Conversely, the ACPA-positive group exhibited a significant increase in classical monocyte proportions (p < 0.01) (**Figure 1E**). No significant difference was identified in intermediate monocyte proportions between the two groups (**Figure 1E**).

### Macrophages and fibroblasts are increased in ACPA-positive synovial tissue

**Figure 2:**
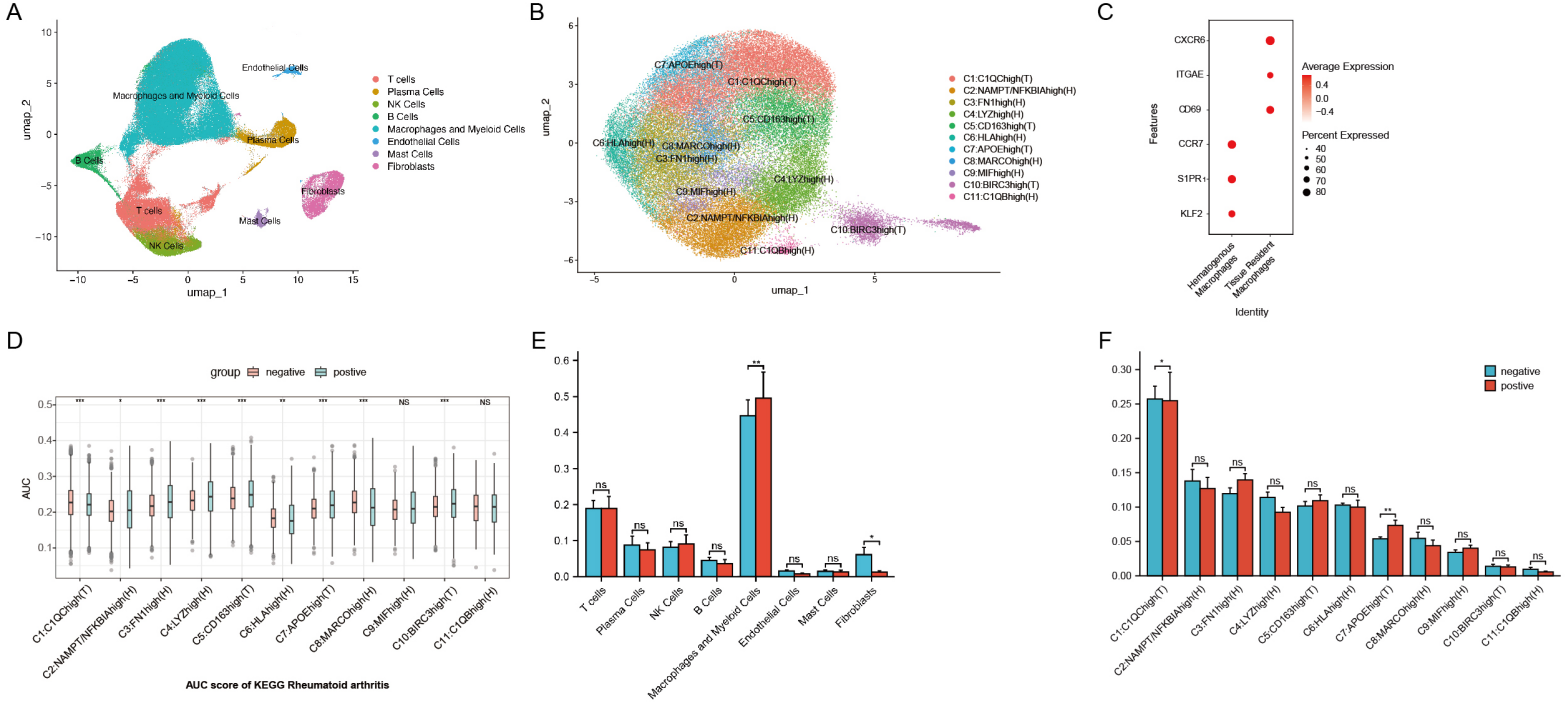
**(A)** UMAP plot of scRNA-seq data from synovial cells. **(B)** UMAP plot of macrophage subtypes from synovial cells. **(C)** Markers for hematogenous macrophages and tissue-resident macrophages. **(D)** AUCell activity scoring of the 11 macrophage subtypes in the KEGG Rheumatoid Arthritis pathway. **(E)** Wilcoxon test comparing synovial cell populations between ACPA+ and ACPA− groups. **(F)** Wilcoxon test comparing macrophage subpopulations from synovial cells between ACPA+ and ACPA− groups. (P-values are expressed as follows: * p ≤ 0.05, ** p ≤ 0.01, *** p ≤ 0.001, and NS indicates no significance.).

Recognizing synovial inflammation as a hallmark of RA, dimensionality reduction and clustering analysis were performed on scRNA-seq data from synovial cells. This approach identified eight distinct cell populations within synovial tissue: T cells, plasma cells, NK cells, B cells, macrophages and myeloid cells, endothelial cells, mast cells, and fibroblasts (**Figure 2A**, **Supplementary Figure 1C**). Each cell type was annotated based on classical markers, selected for their established involvement in RA-related inflammation and immune response. Specifically, T cells were characterized by CD3D and CD3E expression, B cells by CD19 and CD79A, and plasma cells by markers such as MZB1 and IGLC2. Macrophages and myeloid cells showed high CD68 and LYZ levels, while endothelial cells were identified by PECAM1 and VWF. Mast cells were marked by TPSAB1, and fibroblasts by ACTA2 and DCN expression. These cell types are well-documented contributors to the inflammatory cascade and tissue damage observed in RA, providing insights into the cellular landscape of synovial inflammation. Using the Wilcoxon test, immune cell proportions were compared between patients with ACPA+ RA and those with ACPA− RA. Results demonstrated a statistically significant increase in macrophages and myeloid cells (p < 0.01) and fibroblasts (p < 0.05) in the ACPA+ group (**Figure 2E**), aligning with the roles of macrophages and fibroblasts in sustaining inflammation and facilitating joint destruction in RA. No significant differences were found for T cells, plasma cells, NK cells, B cells, endothelial cells, or mast cells (**Figure 2E**).

### Identification of 11 distinct macrophage subtypes with differential roles in RA

Focusing on macrophages, which are central to chronic inflammation, tissue destruction, and immune dysregulation in RA, further dimensionality reduction and clustering analysis identified 11 distinct macrophage subtypes based on gene expression profiles: C1:C1QChigh(T), C2:NAMPT/NFKBIAhigh(H), C3:FN1high(H), C4:LYZhigh(H), C5:CD163high(T), C6:HLAhigh(H), C7:APOEhigh(T), C8:MARCOhigh(H), C9:MIFhigh(H), C10:BIRC3high(T), and C11:C1QBhigh(H) (**Figure 2B**, **Supplementary Figure 1D**). These subtypes reflect macrophage populations with diverse roles in RA. Here, T denotes tissue-resident macrophages, which sustain local inflammation in synovial tissue, while H represents hematogenous macrophages, recruited from the bloodstream in response to inflammatory signals. Tissue-resident macrophages were identified by CXCR6, ITGAE, and CD69 markers, while hematogenous macrophages were marked by S1PR1, KLF2, and CCR7, following marker definitions from prior studies (**Figure 2C**) (32–34).

### Specific macrophage subtypes are enriched in ACPA-negative and ACPA-positive RA

KEGG enrichment analysis on differentially expressed genes across 11 macrophage clusters revealed that genes downregulated in ACPA-positive samples (i.e., upregulated in ACPA-negative samples) were enriched in RA-related subgroups, particularly clusters C1 and C7. These genes were associated with immune pathways such as Th17, Th1, and Th2 cell differentiation. Conversely, ACPA-positive samples showed lower counts and higher p-values in the upregulated differentially expressed genes for C1 and C7, indicating less enrichment compared to ACPA-negative samples (**Supplementary Figures 2A, B**).

**Figure 3:**
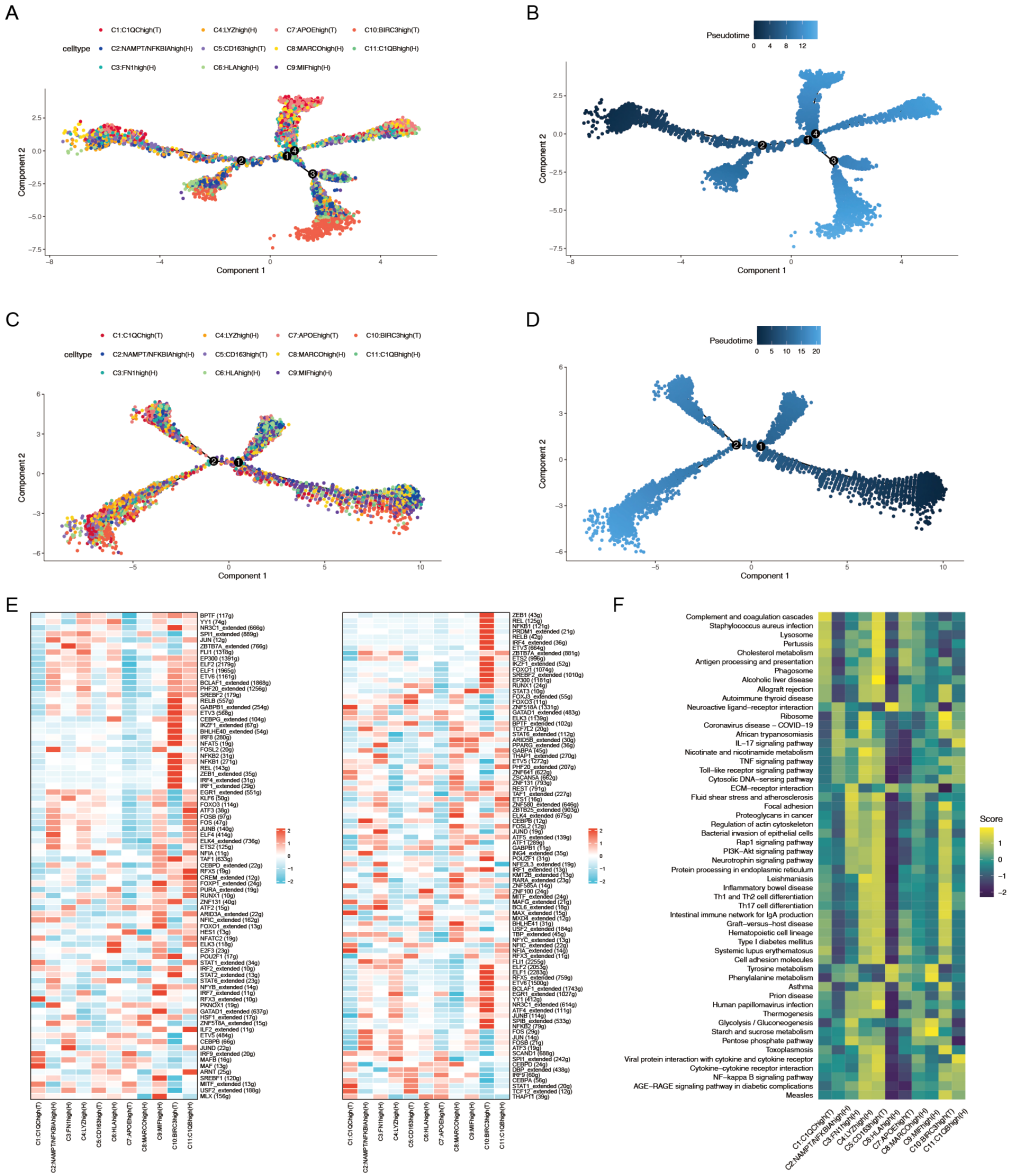
**(A)** Pseudotime trajectory analysis of macrophage subtypes in patients with ACPA− RA. **(B)** Pseudotime progression of macrophage subtypes in patients with ACPA− RA. **(C)** Pseudotime trajectory analysis of macrophage subtypes in patients with ACPA+ RA. **(D)** Pseudotime progression of macrophage subtypes in patients with ACPA+ RA. **(E)** Heatmap of transcription factor activity in ACPA− (left panel) and ACPA+ (right panel) RA macrophages. **(F)** GSVA analysis of macrophage subpopulations.

GSVA identified several key pathways in C1 and C7, including complement and coagulation cascades, allograft rejection, alcoholic liver disease, phagosome, antigen processing and presentation, cholesterol metabolism, pertussis, lysosome, and Staphylococcus aureus infection (**Figure 3F**). Further Wilcoxon test analysis indicated a significant increase in the proportion of C1 macrophages in the ACPA-negative group (p < 0.05), suggesting these cells contribute to local inflammation and synovial hyperplasia in ACPA-negative RA. In contrast, a significant decrease in C7 proportions was observed in the ACPA-negative group (p < 0.01), suggesting that C7 macrophages may have a regulatory or protective function that is diminished in ACPA-positive RA (**Figure 2F**).

AUCell activity scoring for rheumatoid arthritis pathways in the KEGG database across the 11 macrophage subtypes revealed distinct activity patterns between ACPA-positive and ACPA-negative groups. Specifically, C1, C2, C5, C6, C7, and C10 exhibited significantly higher activity scores in the ACPA-positive group, whereas C1 and C8 had notably higher activity in the ACPA-negative group. These results suggest that C1 and C8 may play pivotal roles in ACPA-negative RA, while other subtypes are more active in ACPA-positive RA (**Figure 2D**).

### ACPA-negative RA macrophages display more complex developmental trajectories

To elucidate the dynamic roles of macrophage subtypes in RA progression and immune responses, pseudotime trajectory analysis was conducted on 11 macrophage subtypes to investigate their developmental paths (**Figures 3A–D**). Separate analyses were performed for macrophages from ACPA− RA and ACPA+ RA individuals. Results indicated that macrophages in ACPA− RA exhibited a more intricate developmental trajectory, forming four distinct branches (**Figure 3A**), whereas ACPA+ RA macrophages formed only two branches (**Figure 3C**). This suggests greater diversity in developmental and activation processes among macrophages in ACPA− RA, possibly reflecting increased heterogeneity in macrophage function compared to ACPA+ RA.

Within ACPA− RA, certain macrophage subtypes displayed distinct patterns along the developmental path. Subtypes C10(T) and C11(H), for instance, appeared primarily in early developmental stages, indicating a role in initial macrophage activation or differentiation. In contrast, C1(T) and C7(T) spanned both early and late stages but were absent from intermediate stages, suggesting that these subtypes may have specialized roles at the onset and resolution phases of the macrophage lifecycle, potentially involved in initiating and resolving inflammation. In ACPA+ RA, macrophage subtypes were more uniformly distributed along the trajectory, indicating less developmental complexity, which may reflect a more sustained and homogeneous inflammatory response in ACPA+ RA. The increased developmental complexity and unique pathway involvement in ACPA− RA highlight a higher degree of macrophage heterogeneity, which could contribute to the variable clinical presentation and disease progression observed in ACPA− RA.

To elucidate the biological relevance of pseudotime-related changes, KEGG enrichment analysis was performed on genes associated with pseudotime trajectories for ACPA+ RA and ACPA− RA macrophages. Both groups shared 141 pathways, including key inflammatory and RA-related pathways such as Rheumatoid arthritis and Osteoclast differentiation, which are fundamental to RA pathology (**Supplementary Table 1**).

Distinctly, ACPA− RA macrophages were enriched in seven pathways, including Complement and coagulation cascades, Antifolate resistance, and Glycosphingolipid biosynthesis – ganglion series. These pathways suggest specific roles in the development and progression of ACPA− RA (**Supplementary Table 1**). Enrichment in the Complement and coagulation cascades pathway implies a role in heightened inflammation and immune activation, potentially exacerbating joint damage. Antifolate resistance indicates an altered response to treatments such as methotrexate, suggesting the potential need for alternative therapeutic strategies in patients with ACPA− RA. Furthermore, enrichment in Glycosphingolipid biosynthesis suggests unique lipid metabolism influencing macrophage activity and immune regulation, further distinguishing ACPA− RA from ACPA+ RA. These pathways underscore critical biological differences that may impact both treatment response and disease progression in ACPA− RA.

Conversely, ACPA+ RA macrophages were enriched in 22 unique pathways, including key signaling pathways such as Sphingolipid signaling pathway, JAK-STAT signaling pathway, mTOR signaling pathway, and Adipocytokine signaling pathway (**Supplementary Table 1**). These pathways are pivotal in immune regulation and inflammation, with their enrichment in ACPA+ RA macrophages pointing to distinct molecular mechanisms underlying the more aggressive disease phenotype commonly observed in patients with ACPA+ RA.

### More extensive transcription factor networks in ACPA-positive RA macrophages

To further elucidate the gene regulatory mechanisms underlying these differences, SCENIC analysis was conducted to infer TF regulatory networks. This analysis identified 80 active TFs regulating macrophage subtypes in ACPA− RA and 90 active TFs in ACPA+ RA (**Figure 3E**). Notably, 43 TFs were shared between the two groups, indicating common regulatory mechanisms in macrophage activation across both ACPA+ and ACPA− RA (**Figure 3E**). However, the number of genes regulated by these shared TFs was greater in ACPA+ RA, suggesting a more extensive and complex gene regulatory network in this group. This expanded network in ACPA+ RA likely reflects a more robust and uniform activation of regulatory pathways, consistent with the severe and sustained inflammatory phenotype frequently observed in patients with ACPA+ RA.

### Gene modules associated with ACPA-negative RA identified by hdWGCNA

To investigate the molecular mechanisms of macrophage subtypes associated with ACPA-negative (ACPA−) RA, high-dimensional weighted gene co-expression network analysis (hdWGCNA) was employed. While traditional WGCNA and other dynamic network analysis tools are effective for bulk RNA-seq data, hdWGCNA provides distinct advantages for high-dimensional single-cell RNA-seq, being optimized to address unique challenges such as data sparsity, high noise levels, and the need for granularity in capturing cell-type-specific networks. Unlike standard WGCNA, hdWGCNA preserves cellular-level data structure, making it well-suited to the complex heterogeneity present in RA macrophage populations.

The hdWGCNA approach enabled the identification of modules of highly co-expressed genes, offering biological insights through enrichment analysis and integration with known pathways. An optimal soft threshold of 7 was chosen to ensure a scale-free network topology, facilitating robust co-expression analysis. Using this threshold, a co-expression network was constructed, identifying seven distinct gene co-expression modules, each representing a unique set of interconnected genes with potential regulatory roles in macrophage function.

**Figure 4:**
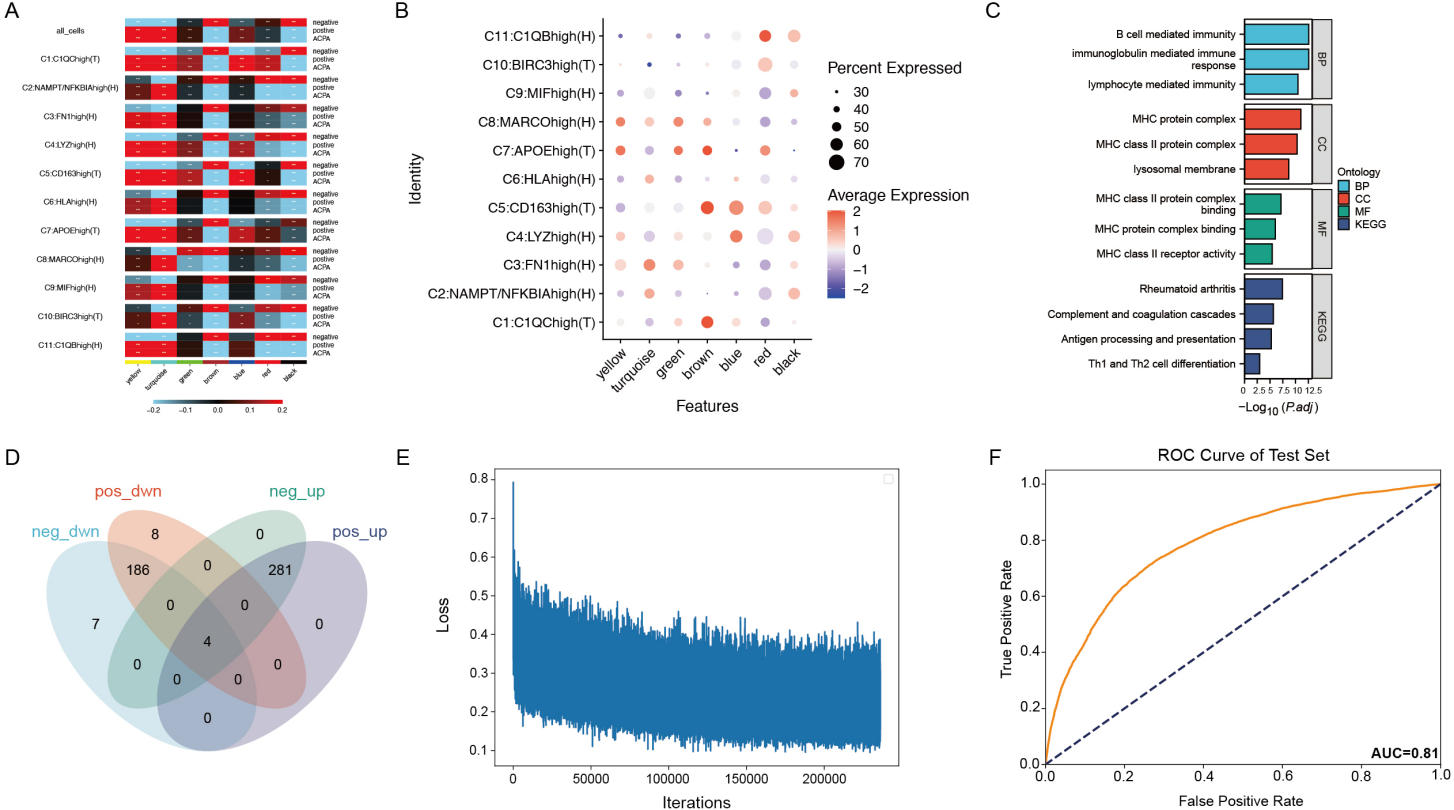
**(A)** Correlation heatmap of seven gene co-expression modules identified by WGCNA in macrophage subtypes. **(B)** Dot plot showing gene expression within the brown module across macrophage subtypes. **(C)** Enrichment analysis of genes in the brown module. **(D)** Venn diagram showing the overlap of 426 genes from the brown module with differentially expressed genes in PBMCs and macrophage subtypes. **(E)** Training loss curve for the deep neural network model distinguishing patients with ACPA− RA from healthy controls. **(F)** ROC curve displaying the neural network model’s performance on the test set.

Correlation analysis between these modules and ACPA+/- RA showed that the brown, red, and black modules were associated with ACPA−, while the yellow, turquoise, and blue modules were linked to ACPA+ (**Figure 4A**, **Supplementary Table 2**). The brown module, in particular, exhibited high expression in macrophage subtypes C1:C1QChigh(T), C5:CD163high(T), and C7:APOEhigh(T) (**Figure 4B**). Enrichment analysis on the brown, red, and black modules revealed that the brown module was enriched in critical immune-related pathways, such as MHC class II-related pathways, Rheumatoid arthritis, Complement and coagulation cascades, Antigen processing and presentation, and Th1 and Th2 cell differentiation (**Figure 4C**).

By intersecting the 426 genes in the brown module with differentially expressed genes in PBMCs and macrophage subtypes, 20 intersecting genes were identified (**Figure 4D**), indicating their differential expression in both PBMCs and macrophage subtypes. Given that anti-cyclic citrullinated peptide (anti-CCP) antibodies serve as an important diagnostic marker for RA but are absent in patients with ACPA− RA, complicating diagnosis relative to ACPA+ RA, these 20 differentially expressed genes were leveraged to construct a neural network model.

### Neural network model distinguishes ACPA-negative RA from healthy controls

Using PBMC expression data and cell-type annotations, a deep neural network was constructed to distinguish patients with ACPA− RA from healthy controls. The data was split into a 70% training set and a 30% test set, with the model trained over 1000 epochs using mini-batch gradient descent (**Figure 4E**). To ensure robustness and prevent overfitting, the ROC curve was evaluated for both training and test sets, achieving an AUC of 0.92 on the training set and 0.81 on the test set. To further validate the robustness and generalizability, five-fold cross-validation was applied, with the average AUC across folds reaching 0.87 and individual AUCs ranging from 0.84 to 0.89. These results indicate stable model performance, supporting the potential clinical application of single-cell transcriptomics for RA diagnostics (**Figure 4F**).

### ACPA-positive RA exhibits stronger macrophage-monocyte communication

Examining PBMC monocyte and macrophage subtype interactions in RA is essential to understanding systemic immune responses that contribute to local joint inflammation and tissue damage. These interactions highlight mechanisms driving chronic inflammation, reveal biomarkers for disease progression, and identify therapeutic targets by isolating specific pathways involved in monocyte-to-macrophage differentiation.

**Figure 5:**
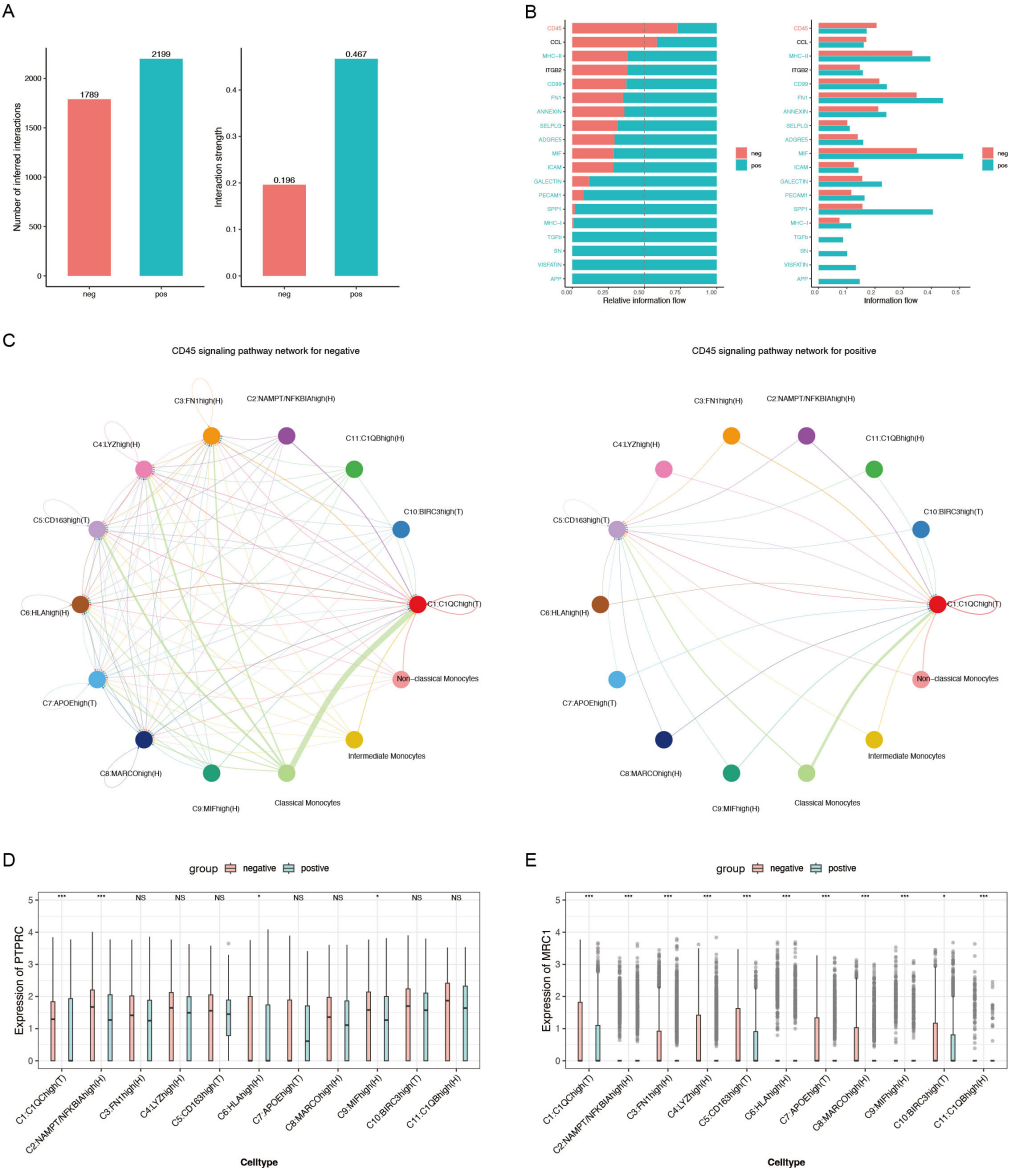
**(A)** Bar chart depicting the number of inferred interactions and interaction strengths. **(B)** Relative information flow of key signaling pathways mediating macrophage-monocyte communication. **(C)** CD45 signaling pathway network for ACPA− (left) and ACPA+ (right) RA. **(D)** Box plot of PTPRC expression, a critical component of the CD45 signaling pathway. **(E)** Box plot of MRC1 expression, another essential component of the CD45 signaling pathway. (P-values are expressed as follows: * p ≤ 0.05, *** p ≤ 0.001, and NS indicates no significance.).

CellChat was utilized to analyze cell communication between monocytes and macrophages in ACPA+ and ACPA− RA. ACPA+ RA showed 2,199 inferred interactions, higher than the 1,789 interactions observed in ACPA− RA. Interaction strength was also significantly higher in ACPA+ RA (0.467) compared to ACPA− RA (0.196) (**Figure 5A**), suggesting that macrophage-monocyte communication in ACPA+ RA is more intense, potentially contributing to the aggressive inflammatory response and severe clinical presentation typically seen in ACPA+ RA.

Regarding relative information flow, CD45 and CCL5 emerged as primary pathways mediating macrophage-monocyte communication in ACPA− RA (**Figure 5B**, **Supplementary Tables 3**, **Supplementary Table 4**). The CD45 pathway was particularly critical for cross-organ communication between classical monocytes and C1:C1QChigh(T) macrophages (**Figure 5C**), indicating its role in macrophage activation and recruitment to inflamed tissues in ACPA− RA. In contrast, fewer interactions were observed between classical monocytes and C1:C1QChigh(T) macrophages in ACPA+ RA, suggesting alternative pathways may drive immune responses in ACPA+ RA.

To further explore, the Wilcoxon test was employed to compare gene expression levels of CD45 pathway mediators, specifically PTPRC and MRC1, between the two groups. PTPRC expression was significantly higher in C1(T) and C9:MIFhigh(H) macrophages in ACPA+ RA (P < 0.001 and P < 0.05, respectively) (**Figure 5D**). This elevated expression of PTPRC, a key component of the CD45 pathway, suggests sustained macrophage activation in ACPA+ RA. Additionally, MRC1 showed significantly higher expression in C1(T) macrophages (P < 0.001) (**Figure 5E**), implying a role in modulating immune responses through alternative pathways in this macrophage subtype.

### Iron-mediated cell communication is prominent in ACPA-negative RA

Previous CellChat analysis suggested that macrophage-monocyte communication in PBMCs might be mediated by the CD45 pathway. Given the importance of cell metabolism in RA pathogenesis—particularly in shaping immune cell functions and inflammatory responses—the role of metabolite-mediated interactions between macrophages and monocytes was considered. Metabolic factors such as lipids and iron play significant roles in RA by influencing cellular energy balance, signaling, and differentiation, thereby driving inflammation and disease progression.

**Figure 6:**
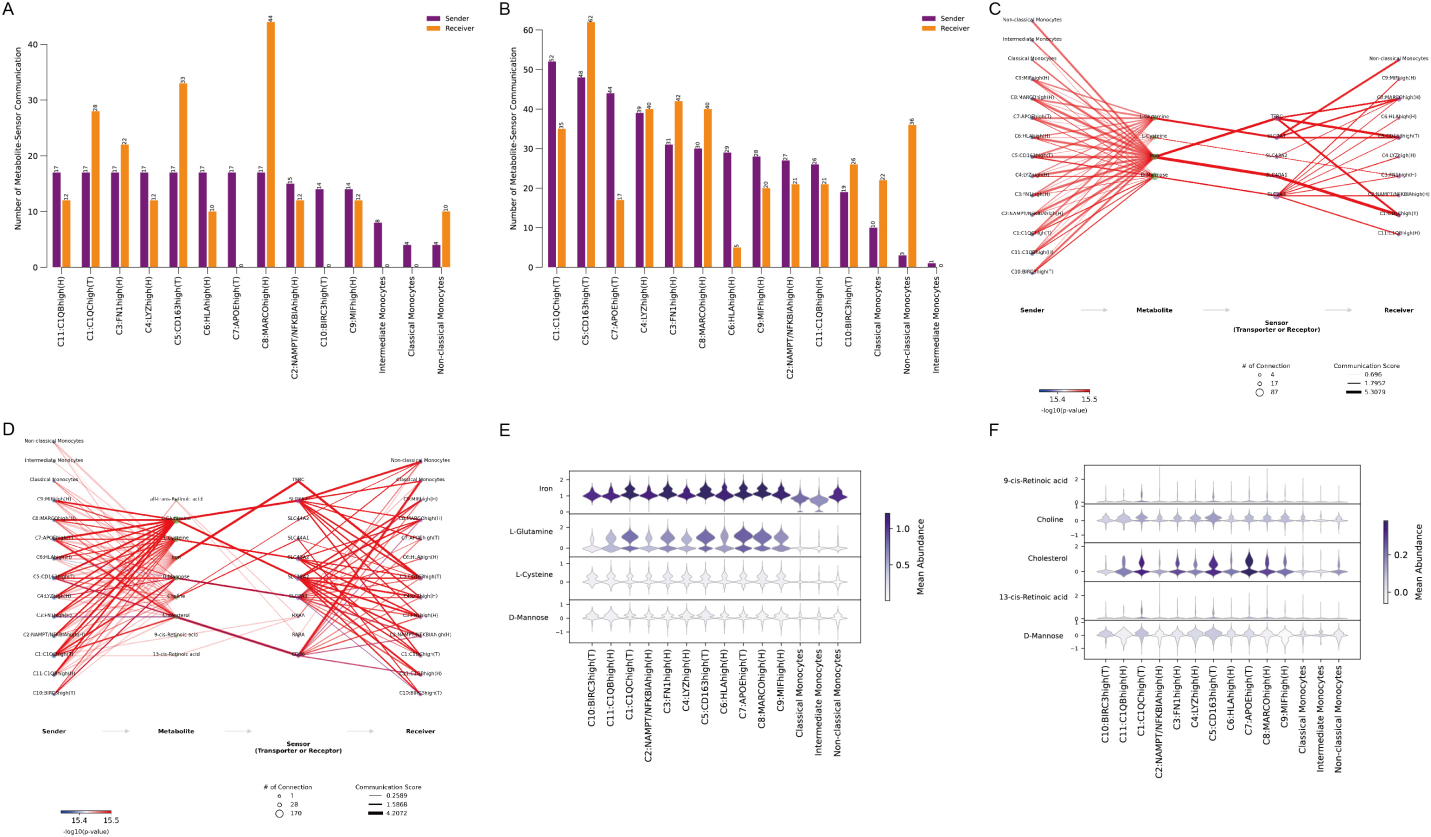
**(A)** Number of metabolite-sensor communication events in ACPA− RA. **(B)** Number of metabolite-sensor communication events in ACPA+ RA **(C)** Metabolite-mediated communication pathways in ACPA− RA. **(D)** Metabolite-mediated communication pathways in ACPA+ RA. **(E)** Violin plots showing the mean abundance of metabolites in ACPA− RA. **(F)** Violin plots showing the mean abundance of metabolites in ACPA+ RA.

To explore this further, MEBOCOST, a Python-based computational tool for inferring metabolite-mediated cell-cell communication from single-cell RNA sequencing data, was employed. Analysis showed a higher number of metabolite-mediated communication events in ACPA+ RA (**Figure 6B**) compared to ACPA− RA (**Figure 6A**). However, focusing on the communication flow from sender metabolite to sensor in the receiver, key interactions were identified in ACPA− RA between monocytes and C1(T) macrophages, as predicted by CellChat. Specifically, the metabolic communication pathways included classical monocytes (sender) – Iron (metabolite) – TFRC (sensor) – C1:C1QChigh(T) macrophages (receiver) and classical monocytes (sender) – Iron (metabolite) – SLC40A1 (sensor) – C1:C1QChigh(T) macrophages (receiver). Additionally, non-classical monocytes displayed similar iron-mediated communication pathways in ACPA− RA (**Figure 6C**, **Supplementary Tables 5**, **Supplementary Table 6**).

In contrast, metabolite-mediated communication between monocytes and macrophages was less prominent in ACPA+ RA (**Figure 6D**), consistent with CellChat findings, indicating that macrophage-monocyte communication may not be as central in ACPA+ RA. This suggests that iron-mediated interactions may be more critical in ACPA− RA, while alternative communication mechanisms could be more relevant in ACPA+ RA.

Visualizing the mean abundance of communication-mediating metabolites revealed higher levels of iron and L-glutamine in ACPA− RA, indicating their roles in macrophage-monocyte interactions (**Figure 6E**). Conversely, cholesterol abundance was higher in ACPA+ RA, pointing to a shift towards lipid-related metabolic pathways in this group (**Figure 6F**). These results underscore distinct metabolic profiles in ACPA− and ACPA+ RA, with iron and glutamine as key mediators in ACPA− RA, while cholesterol may be more influential in the immune response of ACPA+ RA.

### Metabolic flux analysis reveals upregulated pathways in ACPA-negative RA macrophages

To further explore metabolic flux in macrophage subtypes within ACPA− RA, scFEA—a graph neural network model tailored for estimating cell metabolism using scRNA-seq data—was employed. scFEA leverages a reconstructed human metabolic map, utilizing a probabilistic model with flux balance constraints and an optimization solver within a graph neural network to capture the intricate relationships from transcriptomics to metabolomics. This model reflects the non-linear dependencies between enzyme gene expression and reaction rates, using gene expression profiles of macrophage subtypes as input data.

**Figure 7:**
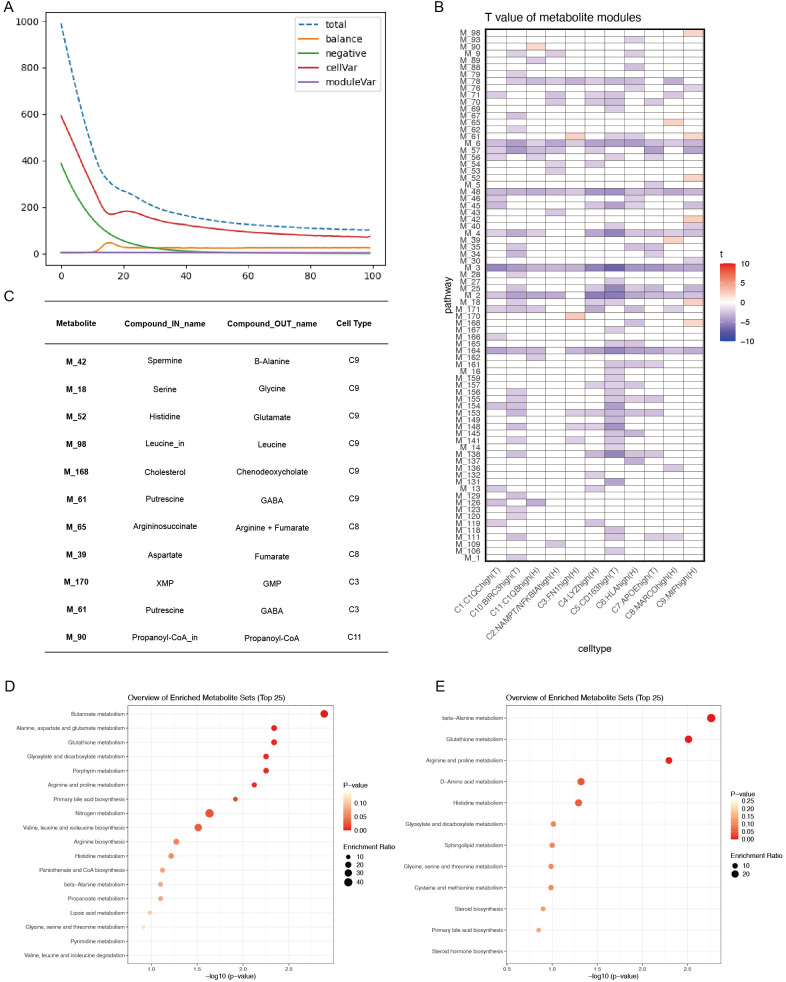
**(A)** Convergence of the loss function during scFEA analysis. **(B)** T-values of metabolite modules across macrophage subtypes, visualized with color codes: red for t-values > 0 (indicating upregulation in the ACPA− group) and blue for t-values < 0 (indicating downregulation in the ACPA− group or upregulation in the ACPA+ group). **(C)** Summary table of the top in-and-out metabolites for significant metabolic modules in ACPA− RA. **(D)** KEGG enrichment analysis of output metabolites from macrophage subtypes. **(E)** KEGG enrichment analysis of input metabolites from macrophage subtypes.

**Figure 7A** shows model convergence through the loss function, confirming its accuracy. Given the continuous and normally distributed output data, the limma package was used to compare ACPA− and ACPA+ RA samples, considering p < 0.05 as statistically significant, with t > 0 indicating upregulation in ACPA− RA and t < 0 indicating downregulation. This analysis identified 11 metabolic modules upregulated in ACPA− RA (**Figures 7B, C**), with each module corresponding to in and out metabolites. Notably, the C9:MIFhigh(H) macrophage subtype exhibited a substantial number of upregulated metabolites. KEGG enrichment analysis of these metabolites was performed using MetaboAnalyst. The input metabolites were predominantly enriched in pathways such as beta-alanine metabolism, Glutathione metabolism, Arginine and proline metabolism, D-amino acid metabolism, and Histidine metabolism (**Figure 7E**). The output metabolites were enriched in pathways including Butanoate metabolism, Alanine, aspartate and glutamate metabolism, Glutathione metabolism, Glyoxylate and dicarboxylate metabolism, Porphyrin metabolism, Arginine and proline metabolism, Primary bile acid biosynthesis, Nitrogen metabolism, and Valine, leucine and isoleucine biosynthesis (**Figure 7D**).

These results suggest that macrophage subtypes, particularly C9:MIFhigh(H), may significantly contribute to RA pathogenesis by promoting key metabolic processes, highlighting distinct metabolic pathways active in ACPA− RA.

## Discussion

This study offers a detailed analysis of the cellular and molecular distinctions of ACPA− RA, particularly focusing on metabolic alterations. scRNA-seq identified unique immune cell compositions, metabolic pathways, and intercellular communication patterns that set ACPA− RA apart from ACPA+ RA. Notably, a marked increase in monocytes, especially classical monocytes, was observed in the PBMCs of patients with ACPA− RA patients compared to patients with ACPA+ RA and healthy controls. This elevation suggests a pivotal role for monocytes in the systemic inflammation that characterizes ACPA− RA. Classical monocytes, known for their potent pro-inflammatory cytokine production and their capacity to differentiate into macrophages and dendritic cells, likely contribute significantly to disease pathology. While previous studies have reported elevated monocyte levels in patients with RA (35), our findings emphasize their increased presence specifically in ACPA− RA, indicating a subtype-specific inflammatory mechanism. This suggests that therapies aimed at monocyte recruitment or activation might be particularly beneficial. Monocyte-targeted interventions, such as inhibitors of monocyte chemoattractant proteins or their receptors, may hold promise for reducing systemic inflammation in ACPA− RA. Additionally, therapies that modulate monocyte differentiation into pro-inflammatory macrophages could help in slowing disease progression in these patients.

In synovial tissue, macrophages from patients with ACPA− RA demonstrated greater heterogeneity and more complex developmental trajectories, forming four distinct branches in pseudotime analysis compared to only two branches in ACPA+ RA. This increased heterogeneity suggests varied activation and differentiation processes, potentially leading to diverse disease courses and therapeutic responses. Notably, macrophages in ACPA− RA were enriched in distinct metabolic pathways, including complement and coagulation cascades, antifolate resistance, and glycosphingolipid biosynthesis. The complement and coagulation cascades are central to immune responses and inflammation. Within the RA context, the complement system contributes to synovial inflammation by promoting opsonization, chemotaxis, and membrane attack complex formation, which drives tissue damage (36). The activation of the coagulation cascade leads to thrombin generation and fibrin deposition in the synovium, intensifying inflammation and encouraging pannus formation (37). These processes establish a pro-inflammatory environment that supports the infiltration and activation of immune cells, such as macrophages and T cells, thereby sustaining joint destruction. Targeting components of the complement and coagulation pathways could thus be a promising therapeutic approach in ACPA− RA, potentially reducing synovial inflammation and preventing joint damage.

The enrichment of metabolic pathways in ACPA− RA macrophages emphasizes the critical role of altered metabolism in disease pathogenesis. The upregulation of complement and coagulation cascades, for example, may intensify inflammatory responses, as components of these pathways act as chemoattractants and immune cell activators (38). Antifolate resistance presents potential treatment challenges, given that methotrexate, a folate antagonist, remains central to RA therapy (39). The identification of glycosphingolipid biosynthesis pathways aligns with evidence that lipid metabolism influences immune cell function and inflammation (40), potentially impacting macrophage activation and cytokine production in ACPA− RA. These insights suggest that therapies targeting metabolic pathways, such as inhibitors of specific enzymes in glycosphingolipid biosynthesis like glucosylceramide synthase, may modulate macrophage function and reduce inflammation in ACPA− RA. Addressing antifolate resistance with alternative disease-modifying antirheumatic drugs (DMARDs) or combination therapies could further enhance treatment efficacy. Some inhibitors, like eliglustat, are already approved for Gaucher disease (41), though their viability in RA requires further investigation. Elucidating macrophage metabolic dependencies in ACPA− RA could guide the development of selective therapies that target pathogenic immune cell subsets while sparing normal immune function.

scFEA further identified 11 upregulated metabolic modules in ACPA− RA macrophages, enriched in pathways like beta-alanine and glutathione metabolism. Beta-alanine metabolism is linked to carnosine synthesis, an antioxidant dipeptide that can modulate inflammatory responses (42). Glutathione metabolism is essential for redox balance and cellular protection against oxidative stress, which is elevated in RA (43). The pronounced role of the C9:MIFhigh(H) macrophage subtype in driving these metabolic pathways suggests that specific macrophage populations contribute to the metabolic reprogramming seen in ACPA− RA. Targeting these metabolic pathways could provide novel therapeutic approaches. Enhancing glutathione levels or modulating its metabolism might alleviate oxidative stress and inflammation in the synovial environment. N-acetylcysteine, a glutathione precursor, is already used clinically for other indications and could be repurposed for RA treatment (44). Similarly, interventions targeting beta-alanine metabolism and carnosine synthesis could influence macrophage activation or cytokine production (45). Identifying the C9:MIFhigh(H) macrophage subtype as a driver of these metabolic alterations highlights it as a potential therapeutic target. Agents that inhibit MIF (macrophage migration inhibitory factor) or its downstream signaling could reduce inflammation and tissue damage in patients with ACPA− RA (46, 47).

Our cell-cell communication analysis revealed that macrophage-monocyte interactions in ACPA− RA are primarily mediated by CD45 and CCL5 signaling pathways. CD45, a receptor tyrosine phosphatase encoded by PTPRC, is critical for T-cell and B-cell receptor signaling and can modulate macrophage activation (48). The involvement of CD45 and its ligands, such as MRC1, suggests a shift in immune regulation in ACPA− RA. Additionally, metabolite-mediated communication analysis highlighted significant engagement of iron-mediated pathways. Elevated iron and L-glutamine levels in patients with ACPA− RA point to a pivotal role for iron metabolism in immune cell interactions. Iron can drive macrophage polarization towards a pro-inflammatory phenotype (49), while the increased abundance of L-glutamine, a key amino acid for immune cell proliferation and function, underscores the metabolic demands of activated immune cells in ACPA− RA (50). Therapeutically, targeting the CD45 pathway may offer a means to modulate macrophage activation and reduce inflammation (48). CD45 inhibitors, already explored in other inflammatory conditions, hold potential for repurposing in RA (51). Modulating iron metabolism presents another promising strategy; iron chelators or agents that regulate iron homeostasis could influence macrophage polarization and attenuate pro-inflammatory responses (52). Additionally, interventions that restrict glutamine availability or inhibit glutamine metabolism could limit immune cell proliferation and activation, providing another therapeutic approach (50). Such strategies may be especially beneficial for patients with ACPA− RA, who often respond suboptimally to standard treatments.

These metabolic alterations may underlie the distinct clinical features of ACPA− RA. Unlike ACPA+ RA, typically associated with more severe joint damage and systemic manifestations, ACPA− RA may follow a different trajectory due to these metabolic distinctions. Our findings support previous research suggesting that metabolic reprogramming of immune cells is a hallmark of autoimmune diseases (53). Future studies should aim to validate these metabolic pathways as biomarkers for disease progression and treatment response in ACPA− RA. Longitudinal studies examining metabolic profile changes pre- and post-therapy could further clarify their clinical utility. Additionally, clinical trials evaluating agents that target these metabolic pathways could assess their efficacy and safety in ACPA− RA, paving the way for more personalized treatment strategies.

Weighted gene co-expression network analysis (WGCNA) identified key gene modules associated with ACPA− RA, particularly the brown module, which is enriched in immune-related pathways. Intersecting genes from this module with differentially expressed genes in PBMCs and macrophage subtypes pinpointed 20 genes differentially expressed in both compartments. Notably, genes such as HLA-DRA, CD74, and FCER1G, which are involved in antigen presentation and immune activation, emerged as potential biomarkers or therapeutic targets. Modulating HLA-DRA and CD74 could influence antigen presentation to T cells, potentially mitigating autoimmune responses (54). Small molecules or antibodies targeting these proteins could be developed, though this would require extensive research and development. A neural network model utilizing these genes was constructed, effectively distinguishing patients with ACPA− RA from healthy controls with an area under the curve (AUC) of 0.81. This outcome underscores the potential of integrating scRNA-seq data with machine learning to enhance ACPA− RA diagnosis, especially given the absence of specific serological markers in these patients. Early and precise diagnosis is essential for initiating timely treatment and improving patient outcomes. To advance these results into clinical practice, further validation of the neural network model is required. Prospective studies with larger, independent cohorts are necessary to confirm its diagnostic accuracy and reliability. Additionally, integrating this model into clinical workflows would necessitate developing accessible assays or platforms to measure the identified genes, potentially through targeted PCR panels or immunoassays. Considerations around regulatory approval and cost-effectiveness would also be essential. Ultimately, this approach holds promise for enabling earlier diagnosis and more personalized treatment strategies for patients with ACPA− RA.

### Limitation

While this study provides valuable insights, certain limitations exist. The cross-sectional design precludes evaluation of temporal changes in immune cell metabolism and function. Future studies with larger, longitudinal cohorts are needed to validate these findings and further investigate the therapeutic potential of targeting metabolic pathways in ACPA− RA.

## Conclusion

In conclusion, this study underscores the significant role of altered metabolism in ACPA− RA pathogenesis. The identification of distinct immune cell compositions, metabolic pathways, and intercellular communication patterns enhances understanding of the disease and suggests new avenues for therapeutics targeting metabolic processes. By pinpointing specific metabolic pathways and immune cell interactions unique to ACPA− RA, these findings highlight potential biomarkers and therapeutic targets that could support the development of more effective, personalized treatments. Future research should focus on clinically validating these targets and examining their impact on patient outcomes. Targeting the metabolic reprogramming of immune cells, particularly macrophages, may enable the creation of precise interventions aimed at modulating inflammation and improving clinical outcomes for patients with ACPA− RA.

## Data Availability

The original contributions presented in the study are included in the article/**Supplementary Material**. Further inquiries can be directed to the corresponding author.

## Supplementary material

Supplementary Figure 1
**(A)** Top 3 markers of cell types from PBMC. **(B)** Expression of CD14 and CD16 in monocytes from PBMC. **(C)** Top 3 markers of cell types from STMCs. **(D)** Top 3 markers of cell types in macrophage subpopulations from STMCs.

Supplementary Figure 2
**(A)** Enrichment analysis of DEGs upregulated in ACPA+ macrophage subpopulations. **(B)** Enrichment analysis of DEGs upregulated in ACPA− macrophage subpopulations.

Supplementary Table 1 Pathways enriched by pseudotime-related gene intersections.

Supplementary Table 2 Genes from WGCNA modules in macrophage populations.

Supplementary Table 3 Cell-cell communications between STMC macrophages and PBMC monocytes in patients with ACPA− RA.

Supplementary Table 4 Cell-cell communications between STMC macrophages and PBMC monocytes in patients with ACPA+ RA.

Supplementary Table 5 Metabolite-mediated cell-cell communications between STMC macrophages and PBMC monocytes in patients with ACPA− RA.

Supplementary Table 6 Metabolite-mediated cell-cell communications between STMC macrophages and PBMC monocytes in patients with ACPA+ RA.
