# Supplementary figures and images for: Metabolic reprogramming and macrophage expansion define ACPA-negative rheumatoid arthritis: insights from single-cell RNA sequencing
Source: Front Immunol. 2025 Jan 3;15:1512483. doi: 10.3389/fimmu.2024.1512483 (PMC11739280; doi:10.3389/fimmu.2024.1512483)

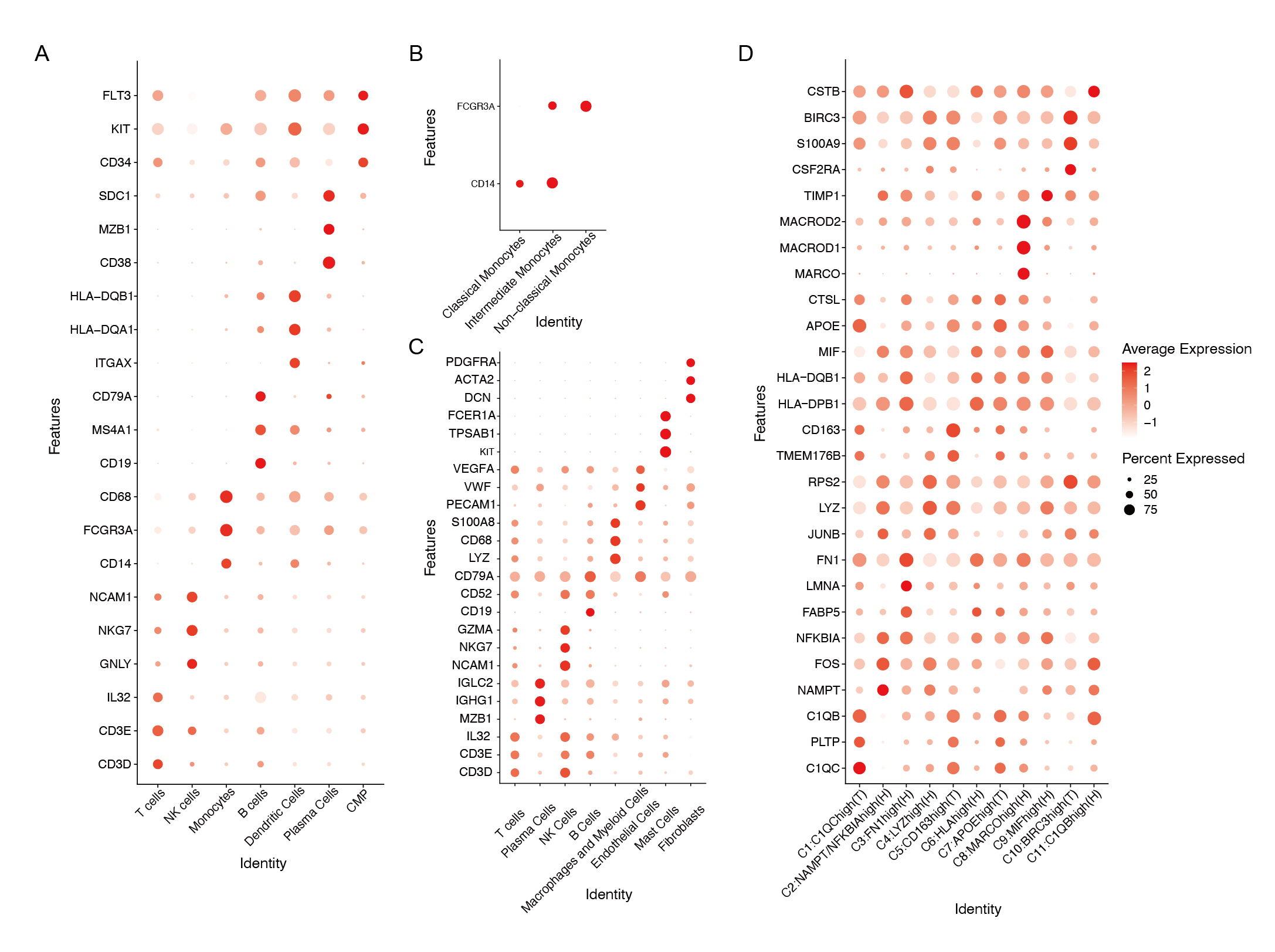

Supplement: Supplementary Figure 1 — (A) Top 3 markers of cell types from PBMC. (B) Expression of CD14 and CD16 in monocytes from PBMC. (C) Top 3 markers of cell types from STMCs. (D) Top 3 markers of cell types in macrophage subpopulations from STMCs. [file Image1.jpeg]

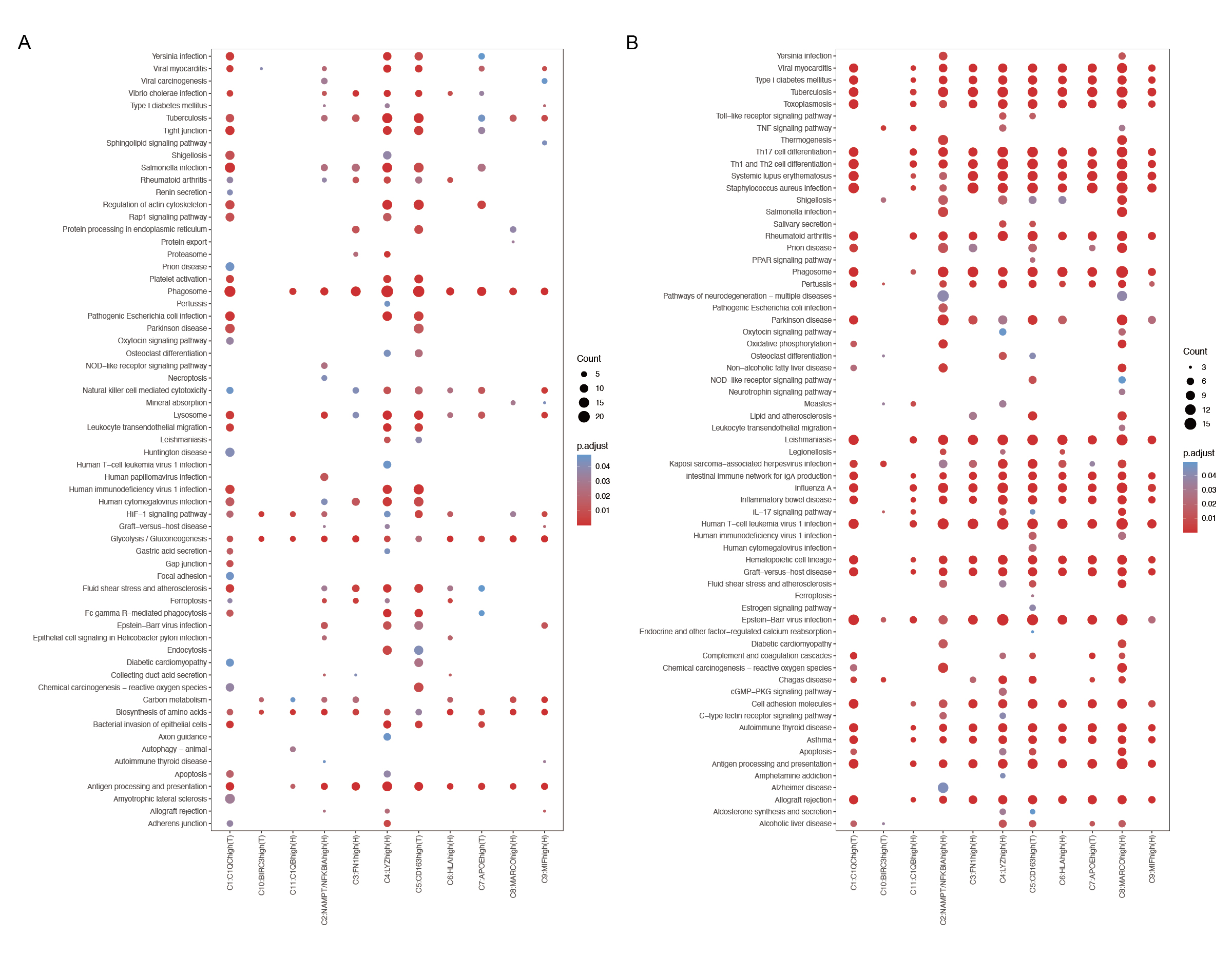

Supplement: Supplementary Figure 2 — (A) Enrichment analysis of DEGs upregulated in ACPA+ macrophage subpopulations. (B) Enrichment analysis of DEGs upregulated in ACPA− macrophage subpopulations. [file Image2.jpeg]
